# Pediatric acute leukemia mimicking osteomyelitis: clinical and MRI features

**DOI:** 10.3389/fonc.2026.1872357

**Published:** 2026-07-14

**Authors:** Stefano Malvestiti, Edoardo Muratore, Simon Wiedemann, Kristin Goller-Bruchmann, Michelangelo Baldazzi, Tamara Abdul-Nour, Brigitte Strahm, Roland Elling, Christian Flotho, Tobias Feuchtinger

**Affiliations:** 1 Department of Pediatrics and Adolescent Medicine, Division of Pediatric Hematology and Oncology, University Medical Center, Faculty of Medicine, University of Freiburg, Freiburg, Germany; 2 Pediatric Hematology and Oncology, IRCCS Azienda Ospedaliero-Universitaria di Bologna, Bologna, Italy; 3 Department of Diagnostic and Interventional Radiology, University Medical Center Freiburg, Faculty of Medicine, University of Freiburg, Freiburg, Germany; 4 Pediatric and Adult CardioThoracic and Vascular, Oncohematologic and Emergency Radiology Unit, IRCCS Azienda Ospedaliero-Universitaria di Bologna, Bologna, Italy; 5 Department of General Pediatrics, Adolescent Medicine and Neonatology, University Medical Center, Faculty of Medicine, University of Freiburg, Freiburg, Germany; 6 Department of General Pediatrics, Adolescent Medicine and Neonatology, Division of Pediatric Rheumatology and Clinical Infectious Disease, University Medical Center, Faculty of Medicine, University of Freiburg, Freiburg, Germany

**Keywords:** acute lymphoblastic leukemia, acute myeloid leukemia, ALL, AML, magnetic resonance imaging, MRI, osteomyelitis, pediatrics

## Abstract

**Background:**

Pediatric acute leukemia (AL) typically presents with symptoms of cytopenias, organomegaly, and fever. In some instances, however, musculoskeletal pain may be the predominant—occasionally the sole—initial symptom, leading to misdiagnoses such as osteomyelitis.

**Methods:**

We conducted a retrospective, multicenter, observational, cohort study including children and adolescents (<18 years) diagnosed with acute lymphoblastic leukemia (ALL) or acute myeloid leukemia (AML) between 2017 and 2024 who initially presented with osteomyelitis-like clinical and/or radiological features. Clinical presentation, laboratory findings, MRI characteristics, and time to leukemia diagnosis were systematically analyzed and compared with the available literature.

**Results:**

Six patients were included (four ALL, one ALL relapse, and one AML). All patients presented with musculoskeletal pain; at first evaluation, fever was present in 50% of the individuals. Initial peripheral blood counts were normal in four and only mildly abnormal in two cases. Inflammatory markers and lactate dehydrogenase were elevated in five and four patients, respectively. MRI demonstrated multifocal or diffuse bone marrow signal alterations in all but one case. The median interval from first presentation to leukemia diagnosis was 17 days (range, 1–53). Features observed in patients with delayed diagnosis included transient clinical response to antibiotic therapy, lack of hematologic abnormalities, and multifocal MRI involvement.

**Conclusion:**

Pediatric AL can mimic osteomyelitis clinically, hematologically, and radiologically. Awareness of clinical red flags and suspicious MRI patterns may prompt earlier consideration of an underlying hematologic malignancy and reduce diagnostic delay.

## 1 Introduction

Acute leukemia (AL) is the most common malignancy in children and adolescents worldwide. It is broadly classified into acute lymphoblastic leukemia (ALL) and acute myeloid leukemia (AML), which account for approximately 80% and 15%–20% of pediatric cases, respectively (1). Establishing the initial diagnosis can be challenging because hematologic abnormalities and typical clinical findings such as pallor, bruising/unusual bleeding, fever, or organomegaly do not always dominate the early clinical presentation. Patients may instead first exhibit nonspecific symptoms that resemble common, non-malignant conditions—for example, musculoskeletal complaints are reported in up to 30% of children with acute leukemia, occurring more frequently in ALL than in AML (2). Musculoskeletal pain may be the predominant—occasionally the sole—initial symptom, which can delay accurate diagnosis and may lead to initial orthopedic or rheumatologic misinterpretations (3, 4).

While several retrospective studies describe arthralgia, arthritis, and limb pain as common early manifestations of pediatric AL, only limited data specifically address its clinical overlap with osteomyelitis (3–7). Magnetic resonance imaging (MRI) is a cornerstone in the evaluation of suspected osteomyelitis (8, 9). At the same time, MRI is sensitive to bone marrow alterations caused by malignant infiltrations, both solid and hematologic (10–12). However, systematic analyses of MRI-based presentations of pediatric AL— particularly those initially suspected as osteomyelitis—remain scarce and are limited to isolated case reports (6, 7, 10, 13, 14).

Therefore, the present study aims to characterize the clinical, laboratory, and MRI features of pediatric patients with AL who initially presented with osteomyelitis-like manifestations and to compare these findings with the cases reported in the literature. This study seeks also to highlight the following major points: (1) pediatric AL can present with musculoskeletal symptoms and laboratory findings mimicking osteomyelitis, (2) bone marrow MRI changes may be visible in some patients with AL several weeks to months before the appearance of peripheral blasts, and (3) MRI findings are nonspecific but, when interpreted with clinical and laboratory evolution, may raise suspicion for leukemia and support timely hematologic evaluation.

## 2 Methods

### 2.1 Study design and setting

This retrospective, multicenter, observational, cohort study was conducted at the University Children’s Hospital Freiburg, Germany, and the University Children’s Hospital Bologna, Italy. The study period spanned January 2017 to December 2024. In both medical centers, eligible patients were identified through institutional clinical databases, ICD-10 diagnostic codes for AL and osteomyelitis, and cross-referencing with radiology reporting systems. The inclusion criteria were as follows: (1) age <18 years at presentation, (2) confirmed diagnosis of ALL or AML, and (3) initial clinical and/or radiological suspicion of osteomyelitis. “Clinical suspicion” was defined as a documented assessment in the electronic medical record by a physician that the clinical and laboratory presentation was most consistent with osteomyelitis, resulting in corresponding diagnostic and/or therapeutic management. “Radiological suspicion” was defined as documentation in the radiology report stating that the MRI findings were compatible with osteomyelitis or that osteomyelitis was a likely differential diagnosis. The exclusion criterion was failure to meet any of the predefined inclusion criteria.

### 2.2 Data collection

Clinical data were collected retrospectively from electronic medical records and included demographic characteristics, symptoms at presentation, physical findings, and treatment prior to leukemia diagnosis. Laboratory parameters comprised complete blood count (CBC), inflammatory markers (C-reactive protein (CRP), erythrocyte sedimentation rate (ESR)), lactate dehydrogenase (LDH), and other routine laboratory values. Time intervals between first presentation, MRI acquisition, and definitive leukemia diagnosis were recorded. Diagnostic delay was defined as the interval between the first medical admission for suspected osteomyelitis and the confirmed diagnosis of AL.

### 2.3 MRI acquisition and evaluation

MRI examinations were performed according to clinical indications using standard pediatric protocols. Sequences included T1-weighted, T2- weighted, fat-suppressed (FS), and contrast-enhanced (CE) imaging as available. Three board-certified pediatric radiologists (SW, KG, and MB) participated in image interpretation. Each MRI study was reviewed in consensus by two pediatric radiologists with attention to bone marrow signal characteristics, lesion distribution (focal vs. multifocal vs. diffuse), soft tissue involvement, and joint effusions; consequently, interobserver agreement was not assessed. The radiologists had access to original MRI reports and were not blinded to prior imaging interpretations.

### 2.4 Literature search and selection

A literature search was performed on PubMed database and Google Search using the following keywords: “hematologic malignancy”, “acute leukemia”, “bone marrow abnormalities”, “magnetic resonance imaging”, “osteomyelitis”, and “infection”. The search included publications issued from January 1, 2005 to December 31, 2025. Case reports, case series, and retrospective cohort studies were considered. Following the electronic database screening, the retrieved literature was manually reviewed and curated to identify illustrative publications reporting cases in which MRI revealed bone marrow abnormalities prior to the diagnosis of acute leukemia or cases initially suspected as osteomyelitis or pyogenic arthritis. The literature review was conducted as a narrative review and was not intended to systematically capture all published cases.

### 2.5 Statistical analysis

Given the exploratory nature and limited sample size, the analyses were descriptive. Continuous variables are reported as medians with range, whereas categorical variables are presented as counts and percentages.

## 3 Results

### 3.1 Patient characteristics

Between January 2017 and December 2024, six pediatric patients were identified and fulfilled the inclusion criteria. The median age at presentation was 4.3 years (range, 1.7–9.7) with equal gender distribution (1:1 = M/F). Five patients were diagnosed with B-cell precursor ALL (BCP-ALL), including one extramedullary ALL relapse manifested as an isolated mandibular bone lesion after hematopoietic stem cell transplantation and one patient with AML. No patient showed central nervous system involvement at diagnosis. The leukemia genetic characteristics are displayed in **Table 1**.

**Table 1:** Patient characteristics, clinical presentation, laboratory parameters at initial presentation, and time latency until leukemia diagnosis.

Patient		#01	#02	#03	#04	#05	#06
Age [years]		1.7	5.6	2.9	8.6	2.2	9.7
Gender		F	M	F	M	F	M
Fever		+	+	+	(+)	(+)	–
Pain	Localization	Abdominal, right knee/leg	Right hip	Abdominal, back, left hip	Mandibular, lower limb	Right hip	Multifocal, mainly lumbosacral
	Frequency	Persistent	Intermittent	Intermittent	Intermittent	Intermittent	Intermittent
Suspected diagnosis		Osteomyelitis	Osteomyelitis	Osteomyelitis	Osteomyelitis	CNO	CRMO
Diagnosis		BCP-ALL	BCP-ALL	BCP-ALL	BCP-ALL relapse	AML	BCP-ALL
CNS		Negative	Negative	Negative	Negative	Negative	Negative
Genetics		Inconclusive	ETV6::RUNX1-fusion	ETV6::RUNX1-fusion	54,XY,+4,+6,+8,+10,+14,+17,+18,+21	46, XX, t(X;11)	46, XX, t(1;19)
Treatment protocol		AIEOP-BFM-ALL-2024-rec.	AIEOP-BFM-ALL-2024-rec.	AIEOP-BFM-ALL-2017	Individualized αCD19-CART	AIEOP-BFM-AML-2020	AIEOP-BFM-ALL-2017
WBC [G/L]		7.0 (4.5–18.8)^a^	6.8 (4.2–13.3)	7.0 (4.5–17.0)	4.3 (3.8–11.9)	8.5 (4.0 –10.0)	12.6 (4.8–12.0)
ANC [G/L]		1.1 (>1.5)	4.0 (>1.5)	1.3 (>1.5)	2.9 (>1.5)	0.7 (>1.5)	6.2 (>1.5)
Hb [g/dL]		6.8 (10–13.7)	10.5 (10.9–14.3)	10.2 (10.3–13.9)	11.9 (11.3–14.7)	10.5 (12.0–15.0)	12.7 (11.2–14.6)
PLT [G/L]		176 (159–532)	363 (177–463)	276 (165–502)	263 (178–426)	377 (150–450)	382 (180–415)
LDH [U/L]		596 (120–300)	575 (120–300)	456 (120–300)	223 (120–300)	n.a.	533 (110–295)
Uric acid [mg/dL]		3.2 (2.4–5.7)	3.1 (3.4–7)	1.9 (2.4–5.7)	3.4 (3.4–7)	n.a.	n.a.
CRP [mg/L]		181.3 (<5.0)	150.0 (<5.0)	13.3 (<5.0)	3.0 (<5.0)	89.0 (<5.0)	22.4 (<5.0)
ESR [mm/h]		170 (<15)	110 (<15)	65 (<15)	n.a.	36 (<15)	55 (<15)
MRI findings		Multifocal	Multifocal	Multifocal	Solitary lesion	Multifocal	Multifocal
MRI Δt [days]		6	38	1	2	21	51
Δt [days]		12	47	1	10	22	53

α, anti; ALL, acute lymphoblastic leukemia; AML, acute myeloid leukemia; ANC, absolute neutrophil count; CNO, chronic nonbacterial osteomyelitis; CRMO, chronic recurrent multifocal osteomyelitis; CRP, C-reactive protein; ESR, erythrocyte sedimentation rate; F, female; Hb, hemoglobin; LDH, lactate dehydrogenase; M, male; n.a., not available; PLT, platelet count; rec., recommendations; WBC, white blood cell count; MRI Δt, time interval between MRI and diagnosis of acute leukemia; Δt, time interval between clinically suspected osteomyelitis and diagnosis of acute leukemia; +, fever was present at initial presentation; (+), fever developed during the course of the disease; –, no fever.

^a^ Age-dependent and gender-dependent normal range values are displayed in brackets.

#### 3.1.1 Clinical presentation and clinical course

Musculoskeletal pain was the leading symptom in all patients (**Table 1**). Pain was initially focal in four cases (#03, #04, #05, and #06) but became multifocal or migratory during follow-up. Three cases (#02, #03, and #05) presented with inability to walk or significant functional impairment. Fever was present at first evaluation in three patients and developed later in two additional cases; one patient remained afebrile (**Table 1**). Three patients had preceding nonspecific symptoms, including upper respiratory infection, abdominal pain, or back pain, resulting in alternative initial diagnoses such as transient synovitis or pneumonia. Persistence (#01 and #03) or recurrence (#02, #04, and #05) of pain despite symptomatic or antimicrobial treatment was observed in five patients and contributed to questioning the differential diagnosis of osteomyelitis. At the time of leukemia diagnosis, four patients (#02, #03, #05, and #06) showed deteriorating clinical condition, and among them, one individual (#06) developed hemorrhagic shock due to severe hematemesis and epistaxis. No patient showed organomegaly or lymphadenopathy at first evaluation. Only case #06 displayed hepatosplenomegaly later at the time of leukemia diagnosis. No patient received corticosteroids prior to the confirmation of leukemia diagnosis.

#### 3.1.2 Laboratory findings

At initial presentation, complete blood counts were normal in two of six patients (33%). Four patients showed mild cytopenias, i.e., normocytic anemia and/or neutropenia (**Table 1**). Inflammatory markers were elevated in five patients, with markedly increased CRP and ESR values (**Table 1**). LDH levels were high in four patients (median: 533 IU/L; range: 223–596 IU/L), while uric acid levels remained within normal limits where assessed. All patients initially received treatment for presumed osteomyelitis or inflammatory bone disease, including empiric antibiotics in five cases (#01–#05) and nonsteroidal anti-inflammatory drugs in one (#06). In several cases (#01, #02, and #05), laboratory abnormalities evolved over time, with transient normalization of inflammatory markers and subsequent development of cytopenias prompting bone marrow examination (**Supplementary Table 2**). Worsening blood counts were indeed detectable at the time of leukemia diagnosis in four individuals (#01: progressive thrombocytopenia, #02: new-onset neutropenia, #05: worsening anemia and neutropenia, and #06: leukocytosis, anemia, and thrombocytopenia). Peripheral blasts were absent in all patients at first evaluation; however, they emerged in four patients (#01, #02, #05, and #06) at the time of leukemia diagnosis (**Supplementary Table 2**).

#### 3.1.3 MRI findings

**Figure 1:**
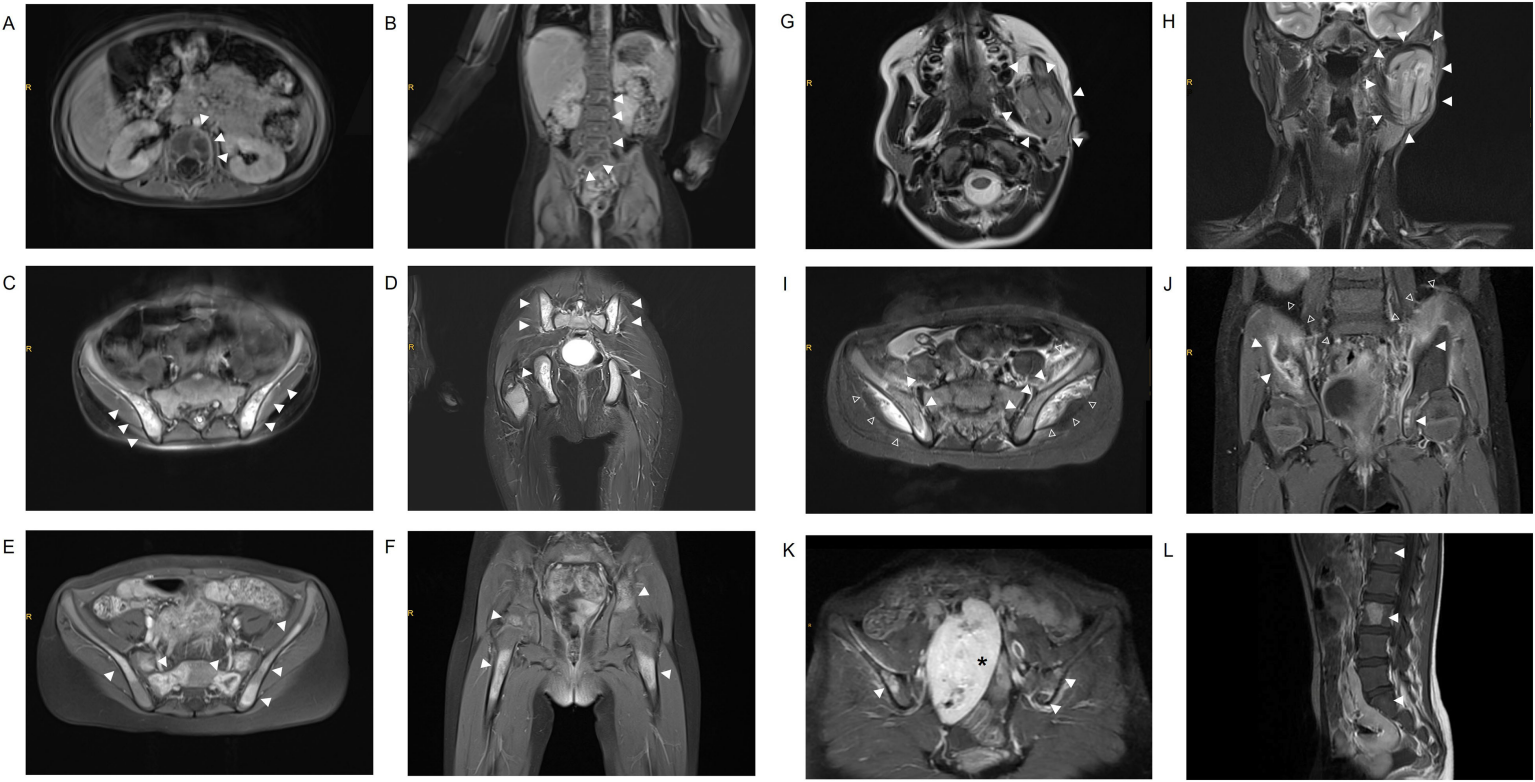
MRI findings in the reported acute leukemia cases (#01–#06). Case #01: axial **(A)** and coronal **(B)** plane of a T1-weighted imaging (WI) showing subtle, focal intraosseous contrast enhancement (◄) in the vertebral bodies L2 **(A, B)**, L3 and L5/S1 **(B)**. Slight adjacent soft-tissue changes were observed in the right gluteal region and at the insertion of the left gluteus maximus. The remainder of the skeleton showed physiologic red marrow signal; extremities, joints, and soft tissues were unremarkable. Case #02 **(C, D)**: axial **(C)** and coronal **(D)** plane of a T2-WI demonstrating multifocal, sheet-like bone marrow edema (◄) involving the medial ilium bilaterally and the pubic bone. Mild adjacent soft-tissue involvement was suspected, including subtle edema in the gluteus maximus muscles. No significant joint effusions were present in the hips, knees, or ankle joints. Case #03 **(E, F)**: axial **(E)** and coronal **(F)** plane of a contrast-enhanced T1-WI showing patchy bilateral enhancement (◄) of the medullary cavity of the ilium, the lateral sacrum, and the proximal femoral diaphysis without involvement of the metaphysis or epiphysis. Case #04 **(G, H)**: axial **(G)** and coronal **(H)** plane of a T2-WI showing an osteodestructive, highly cellular, heterogeneously enhancing mass of the left mandible (◄) extending from the condylar head and coronoid process to the mandibular ramus. The lesion infiltrates large portions of the left masticatory musculature. Case #05 **(I, J)**: axial plane **(I)** of a T2-WI and coronal plane **(J)** of a T1-WI with contrast showing multifocal marrow abnormalities with peripheral edema signal and peripheral contrast enhancement of the medial ilium with central hypointense signal of the medullary cavity (◄) and edema of the adjacent gluteus medius (empty◄) and iliacus muscle. Case #06 **(K, L)**: pelvis-oriented plane **(K)** of a post-contrast fat-suppression (FS) T1-WI and sagittal plane of a post-contrast FS T2-WI **(L)** displaying multiple areas of abnormal signal (◄) involving the L1, L3, L4, and S1 vertebral bodies and with marked contrast enhancement. The largest lesion was at L3, extending through the entire height of the vertebral body and located predominantly along the right anterolateral aspect. Small areas of abnormal signal with pronounced contrast uptake were also present in both iliac wings **(K)**, with a small hypointense avascular area in the left iliac wing reflecting necrosis. The asterisk (*) indicates known nephroptosis.

The median time interval between MRI and leukemia diagnosis was 16.5 days (range, 1–51 days; **Table 1**). MRI demonstrated abnormal bone marrow signals in all patients (**Figure 1**). Multifocal or diffuse marrow involvement was observed in five cases, always extending beyond a single anatomical region. Typical findings included low signal intensity on T1-weighted images (WI) and high signal intensity on fat-suppressed T2-WI, often with contrast enhancement. Lesions most commonly involved the pelvis, sacrum, vertebral bodies, and proximal long bones (**Figures 1A–F, I–L**). Adjacent soft tissue edema was present in four patients (#01, #02, #04, and #05) and joint effusion or synovial enhancement in two (#02 and #05). One patient (#04) showed an osteodestructive mandibular lesion with extensive soft tissue infiltration (**Figures 1G, H**). In four cases (#01, #02, #04, and #05), the MRI reports raised the possibility of malignant marrow infiltration, although infectious or inflammatory etiologies were initially favored clinically. In patient #03, the MRI initially performed for clinically suspected osteomyelitis demonstrated diffuse, bilateral, diaphyseal marrow infiltration, suggesting the presence of acute leukemia, and was therefore essential to initiate hematological analysis.

#### 3.1.4 Diagnostic interval and leukemia diagnosis

The median time interval between first presentation and definitive leukemia diagnosis was 17 days (range, 1–53 days; **Table 1**). The longest delay occurred in a patient initially diagnosed with chronic recurrent multifocal osteomyelitis (CRMO, #06). Definitive diagnosis was established by bone marrow examination in five patients and by biopsy of an extramedullary lesion in one (#04). At diagnosis, peripheral blasts were detectable in four patients, while two were diagnosed despite persistently normal peripheral blood morphology. Patient #04 had an isolated extramedullary relapse. With the exception of case #04, who received anti-CD19 CAR-T cell therapy, all patients were treated according to national disease-specific protocols (**Table 1**), achieved timely complete remission without major treatment-related adverse events, and remain well after completing the therapy (maximal follow-up: 4 years).

#### 3.1.5 Narrative review of available literature

A literature search identified a total of 17 patients—12 children and adolescents and five adults. Each patient underwent MRI following an initial pain-driven presentation in the absence of typical clinical findings of a hematologic disease and prior to establishing the diagnosis of leukemia (**Table 2**). No retrospective studies were identified. All 12 pediatric patients presented with pain and fever as their primary complaints (6, 7, 13, 14). Pain was intermittent in all but one case (7) and was predominantly located in the hip, lower extremities, and lumbar spine. All five adult patients presented with persistent or fluctuating pain, and only one case also developed a fever (15). The majority of the patients had normal CBC at initial presentation; only four exhibited mild hematologic abnormalities including neutropenia, anemia, or leukocytosis. Inflammatory markers (CRP and ESR) and cell lysis parameters (LDH and uric acid) were reported exclusively in the 12 pediatric cases and were elevated in eight and seven patients, respectively. The demographic and laboratory data of the pediatric patients identified in the literature research are provided in **Supplementary Table 1**. MRI findings were available for all 17 patients (**Table 2**). Common features included the presence of bone marrow signal alterations, characterized by a low signal in the T1-WI and a high signal in the T2-WI, consistent with bone marrow edema. Signal abnormalities were frequently diffuse and presented with either focal or multifocal infiltrative patterns. MRI alterations often followed a symmetrical distribution—although asymmetrical changes were observed in occasional cases—and were predominantly located in the pelvic region and the diaphysis of long bones. Soft tissue edema was also reported both in pediatric and adult patients (6, 7, 10). Joint effusion of variable extent was described in two adult (10) and two pediatric individuals (14). With the exception of a single patient, whose outcome was not reported (6), all remaining pediatric leukemia cases achieved complete remission following standard national chemotherapy protocols and were alive at the time of reporting.

**Table 2:** Patients with bone marrow abnormalities in MRI before diagnosis of hematologic malignancy – a review of literature.

Author, Year, Country	Study type	Number of patients / Median age / Gender (M, F)	Initial presentation	Laboratory findings at first presentation§	CBC at leukemia diagnosis	*Time to leukemia diagnosis	AL type
Pediatric literature							
Gomoll et al., 2007, USA (6)	Case report	1 / 3 y / M	Intermittent upper abdominal pain, intermittent fever	LDH and uric acid n.a., ESR >100 mm/h	WBC ↓, Hb ↓, PLT ↓, no peripheral blasts	> 6 wk	BCP-ALL
Goto et al., 2022, Japan (7)	Case report	1 / 1 y / M	Pain in the upper arm, intermittent fever	LDH 344 IU/L, Uric acid and ESR n.a.	CBC w.n.r.	> 12 d	AML
Kato et al., 2011, Japan (13)	Case series	6 / 8 y / 3M, 3F	Intermittent pain (mostly at lower limbs), fever, fatigue	LDH ↑ (3 Pts), LDH w.n.r. (3 Pts), uric acid and ESR n.a., CRP ↑ (3 Pts)	n.a.	> 2 wk	4× BCP-ALL, 1× mature B-ALL, 1× AML
Yoshikawa et al., 2016, Japan (14)	Case series	4 / 3 y / 4M	Alternating migratory joint pain (knee, hip, shoulder, elbow), intermittent fever	WBC ↑ (2 Pts), mild neutropenia (1 Pts), LDH ↑ (3 Pts), LDH w.n.r. (1 Pt), CRP ↑ (4 Pts), ESR and uric acid n.a.	Peripheral blasts (2 Pt); persistent neutropenia (1 Pt); CBC w.n.r., no peripheral blasts (1 Pt)	20 d, 27 d, 40 d, 180 d	4× BCP-ALL
Gronningsaeter et al., 2018, Norway (10)	Case series	4 / 42 y / 2M, 2F	Localized lower limb / hip pain, weight loss, night sweats	Hb ↓ (1 Pt), CRP, ESR, LDH and uric acid n.a.	Hb ↓, PLT ↓, WBC ↑ (1 Pt); CBC w.n.r. (1 Pt)	2.5 y, n.a. (3 Pts)	1× CML, 2× BCP-ALL, 1× APL
Paone et al., 2015, Switzerland (15)	Case report	1 / 83 y / n.a.	Persistent lumbar pain, FUO, inflammatory syndrome	n.a.	Leukoerythroblastic changes in peripheral blood	n.a.	AML
Author, Year, Country	Suspected diagnosis	Imaging Δt^‡^	Imaging type	Antibiotic therapy	Clinical / laboratory improvement on antibiotics	Site of bone involvement	Imaging findings
Pediatric literature							
Gomoll et al., 2007, USA (6)	Sternal osteomyelitis	CT: >1 wkMRI: >1 wk	CT & MRI	+	–	Singular sternal lesion	CT: lytic lesionMRI: soft tissue and bone marrow edema
Goto et al., 2022, Japan (7)	Osteomyelitis	12 d	CT & MRI	+	–	Left humerus	MRI: diffuse focal bone marrow changes with evidence of surrounding soft tissue edema; low signal T1-WI and high signal in T2-WI
Kato et al., 2011, Japan (13)	n.a.	2–6 wk	MRI	n.a.	n.a.	Pelvis and/or lower limbs	MRI: All Pts show diffuse, as well as multifocal/patchy bone marrow changes; low signal T1-WI and high signal in T2-WI
Yoshikawa et al., 2016, Japan (14)	Pyogenic arthritis / osteomyelitis (3 Pts), Rheumatoid arthritis (1 Pt)	2–8 wk	FDG-PET & MRI	+ (3 Pts)– (1 Pt)	+ (2 Pts)– (2 Pts)	Pelvis and/or limbs	MRI: all Pts show diffuse and focal infiltration; joint effusions in 2 Pts; low signal T1-WI (in 4 Pts) and high signal in T2-WI (in 3 Pts)
Adult literature							
Gronningsaeter et al., 2018, Norway (10)	Degenerative osteoarthritis (1 Pt), unspecified malignancy (1 Pt), n.a. (1 Pt), muscular rupture (1 Pt)	30 m (1 Pt), n.a. (3 Pts)	CT & MRI & FDG-PET	n.a.	n.a.	Knees and/or pelvis and/or vertebral bodies	CT: extramedullary lesions (spleen, kidney), (1 Pt)FDG-PET: increased uptake in extramedullary lesions, slight increase throughout the skeleton (1 Pt).MRI: both diffuse focal (1 Pt) and multifocal (3 Pts) bone marrow changes, occasionally with hip joint effusion (2 Pts) and soft tissue edema (1 Pt); low signal T1-WI and high signal in FS T2-WI
Paone et al., 2015, Switzerland (15)	Spondylodiscitis	n.a.	FDG-PET & MRI	n.a.	n.a.	Vertebral bodies Th1 and L1	FDG-PET: patchy increased uptakeMRI: diffuse as well as disseminated focal infiltration of the bone marrow; hypointensity in T1-WI and mixed presentation in T2-WI

§ CBC was within the normal range and no peripheral blasts were detected, whenever not otherwise specified-.

* Whenever not explicitly specified by the authors, “time to leukemia diagnosis” was conservatively approximated by deriving the minimum plausible interval from the timelines reported for each case. ^‡^ Wherever multiple patients were included in the case reports, Δt range is provided. For imaging Δt of the pediatric patients, refer to **Supplementary Table 1**.

AL, acute leukemia; ALL, acute lymphoblastic leukemia; AML, acute myeloid leukemia; APL, acute promyelocytic leukemia; BCP, B-cell precursor; CBC, complete blood count; CML, chronic myeloid leukemia; CT, computed tomography scan; d, day(s); F, female; FDG-PET, fluorodeoxyglucose-positron emission tomography; FS, fat suppressed; FUO, fever of unknown origin; Hb, hemoglobin; L, lumbar; M, male; m, month(s); MRI, magnetic resonance imaging; n.a., not available; PLT, platelets; Pt(s), patient(s); Th, thoracic; WBC, white blood cells; WI, weighted images; wk, week(s); w.n.r., within the normal range; y, year(s); Imaging Δt, time interval between imaging and diagnosis of acute leukemia; +, presence; –, absence.

## 4 Discussion

Osteomyelitis and AL may present a high grade of clinical overlap. Characteristic symptoms and findings at initial presentation can be the same (see **Table 3**), and even when both alternatives are considered from the beginning, establishing the final diagnosis can be challenging. This study aims to reaffirm these diagnostic difficulties and to raise awareness for the rare osteomyelitis-like presentation of AL while contextualizing MRI findings and providing real-world experience.

**Table 3:** Osteomyelitis and acute leukemia: typical clinical and diagnostic findings.

Disease findings	Acute leukemia	Bacterial osteomyelitis	CNO/CRMO
Etiology	Neoplasia (1, 2)	Infection (generally hematogenous) (8, 16)	Autoinflammation (17)
Typical age at diagnosis [years]	<6 (ALL) (1, 2), <2 and >10 (AML) (1, 2)	2–8 with 50% <5 (5, 8)	7–12 (5, 17)
Pain	Chronic–subacute (2, 3), frequently during the night (2, 3), generally not activity-related (2, 3, 5)	Subacute onset (5, 8), exertion-related pain (5, 8)	Timely limited to chronic-recurrent pain (lasting weeks–months) (5, 8, 17)
Predilection areas	Diffuse, bilateral, and frequently changing localization (2, 3, 5), generally involving lower extremities (2, 3, 5), rarely joint effusions (2, 3, 5)	Generally focal localization (8, 9), long bones (25% femur, 25% tibia, 13% humerus, 6% fibula, phalanx, or radius, seldom vertebral bodies) (8, 9)	Unifocal to multifocal (8, 17), generally symmetrical (8, 17), metaphysis, epiphysis, clavicula, mandibula, vertebral bodies, pelvis, lower extremities (8, 17)
Fever	Frequently present (2, 5)	Frequently present (5, 8, 16)	Generally absent (5, 8, 17)
Accompanying symptoms	Unspecific: recurrent infections, bruising, fatigue, paleness, lymph node enlargement (2, 5)	Local redness, swelling, warmth, *functio laesa* (8, 9)	Local redness, swelling, warmth, *functio laesa* (8, 17); comorbid autoimmune/autoinflammatory diseases, e.g., psoriasis, palmoplantar pustulosis, IBD, arthritis (17)
Laboratory studies	Hematological abnormalities, ↑LDH, ↑uric acid (↑inflammation parameters) (2, 5, 18, 19)	↑↑↑inflammation parameters: (ESR), (↑LDH); (hematological abnormalities) (8, 9)	Generally normal CBC, ESR <60 mm/h, CRP <30mg/L (5, 17, 20)
Bacterial isolation	(2, 5)	Blood culture 40% + (5, 8, 16), lesion biopsy 70% + (5, 8, 16)	(5, 17)
Typical MRI findings	Multifocal or diffuse low signal in T1-WI and high signal in fat-suppressed T2-WI (6, 7, 10, 13, 14), commonly affected region: diaphysis (6, 7, 10, 13, 14)	Single or multifocal low signal in T1-WI and high signal in fat-suppressed T2-WI (7, 21, 22), bone marrow edema and cortical destruction (7, 21, 22), post-contrast enhancement of bone marrow, abscess margins, periosteum, and adjacent soft tissue collection (7, 21, 22), commonly affected region: metaphysis, possibly extending to diaphysis or epiphysis (7, 21, 22)	Single or multifocal low signal in T1-WI and high signal in fat-suppressed T2-WI (7, 21, 22), sclerotic, osteolytic, and/or hyperostotic bone lesions, bone marrow, and/or soft tissue edema, synovitis (7, 21, 22), commonly affected region: metaphysis, possibly extending to diaphysis or epiphysis (7, 21, 22)

This table summarizes data from (1–10, 13, 14, 16–22). The level of evidence (23) of the cited studies is listed in **Supplementary Table 3**.

ALL, acute lymphoblastic leukemia; AML, acute myeloblastic leukemia; CBC, complete blood count; CNO, chronic non-bacterial osteomyelitis; CRMO, chronic recurrent multifocal osteomyelitis; IBD, inflammatory bowel disease; MRI, magnetic resonance imaging; T1-WI, T1-weighted imaging; T2-WI, T2-weighted imaging; –, absence.

All patients presented with subfebrile to febrile temperatures and musculoskeletal pain, with the exception of case #06 who was afebrile. Continuous ibuprofen administration for arthralgia could have masked fever in this particular case. Except for cases #02 and #05, the remaining patients in our cohort showed fluctuating pain localization. In line with our observation, migratory and intermittent pain was also described in most of the identified case reports (**Table 2**), a pattern that may be more consistent with an underlying malignancy than with osteomyelitis. At initial evaluation, CBC revealed minor hematologic abnormalities in four cases (#01, #02, #03, and #05). In all patients, neutropenia was initially attributed to a presumed preceding viral infection, while the normocytic anemia was interpreted as anemia of chronic disease. Transient neutropenia following febrile illness can occur in children and generally improves spontaneously. Persistent neutropenia, however, warrants further diagnostic evaluation, particularly considering that up to 10%–15% of children with fever and sustained neutropenia have an underlying malignant disease (24). Normocytic anemia following a prolonged inflammatory episode, such as osteomyelitis, is also frequently reported (16, 25, 26). These considerations provided an initial explanation for the hematologic abnormalities observed in both patients. In cases #01 and #03, the neutrophil count even normalized during the first week of hospitalization. Nonetheless, because bicytopenia is associated with underlying malignant disorders in 5%–10% of cases, as shown by recent pediatric cross-sectional studies (27, 28), its persistence prompted bone marrow evaluation. Interestingly, the combination of anemia and neutropenia appeared to discriminate with high specificity between arthritic disease and ALL and could therefore assist during the diagnostic workup (18). However, those data still have to be confirmed in cases of osteomyelitis and AML. Kato et al. reported three patients without hematologic abnormalities in peripheral blood; in each case, leukemia was diagnosed only after repeated bone marrow biopsies despite initially representative marrow aspirates (13). This observation underscores the importance of constant diagnostic reassessment, whenever leukemia is a plausible differential diagnosis, despite the absence of blasts and CBC abnormalities.

When blood counts are unremarkable, distinguishing osteomyelitis from AL can become more challenging and may require additional analyses. Although LDH and uric acid are sensitive markers for an increased cellular turnover, as commonly observed in AL (3), their specificity is low, and normal values do not exclude malignancy, as illustrated by case #04 and by previously reported patients (**Supplementary Table 1**) (13, 14). Moreover, inflammatory processes of bone and joint structures may also increase the LDH levels, limiting its utility in discriminating osteomyelitis from AL (19). Inflammatory markers were mildly to markedly elevated across our and previous cohorts (**Tables 1**, **2**; **Supplementary Table 1**). It is well known that CRP and ESR can be significantly increased in osteomyelitis and CNO/CRMO (17), thereby lacking specificity for AL, too. As a consequence, it appears mandated for physicians to actively and, where required, repeatedly consider hematologic malignancy as a differential in cases of musculoskeletal pain even in the absence of characteristic laboratory abnormalities.

Although possible, long-lasting pain of several weeks to months is uncommon for AL. Clinicians generally consider more frequent differential diagnoses, such as osteomyelitis, and rapidly resort to MRI as part of the diagnostic workup. In our cohort (particularly in cases #02, #05, and #06), abnormal MRI findings in the bone marrow were detected long before the appearance of any hematologic abnormality or peripheral blasts, without any discernible differences between AML and ALL. Our observations are consistent with previously reported AL cases (**Table 2**) (7, 13, 15, 29). Interestingly, Gronningsaeter et al. reported a case of a 69-year-old patient with bilateral gonalgia and diffuse MRI abnormalities of the knees, which were initially considered as age-related changes. Nearly 2.5 years later, he was diagnosed with chronic myeloid leukemia (10). Retrospectively, the signal aberrations were consistent with bone marrow edema and may have reflected underlying marrow involvement by a hematologic disease, suggesting that in the presence of a leukemic disease—although, in this case, chronic—MRI abnormalities could present long before overt hematologic manifestation. Classically, the bone marrow in AL appears diffusely and uniformly hypointense on T1-WI and hyperintense on fat-suppressed T2-WI. However, the MRI findings might also be very unspecific and show a significant overlap with the typical findings of osteomyelitis (**Figure 1**). In fact, multifocal signal alterations, as well as post-contrast enhancement with soft tissue edema, may present in both AL (**Figure 1**; **Table 2**) and osteomyelitis (13, 14, 30). Occasionally, even marginal contrast enhancement may contribute to misdiagnosis (**Figure 1**). Distinguishing AL from osteomyelitis based on MRI findings is therefore extremely difficult, except in some isolated cases (e.g., case #03 and (10)).

Moreover, osteomyelitis and AL may occur concomitantly (31, 32), representing an important diagnostic confounder. In our literature research, we identified five cases who received antibiotic treatments at presentation; two of them showed at least a transient clinical improvement—however, no information regarding the antibiotic regimen and length of therapy was provided. In our cohort, direct microbiological assessment was performed only in case #04, in which biopsy of the mandibular lesion yielded sterile cultures. Therefore, concomitant osteomyelitis could not be excluded with certainty in all other AL cases. Patients #01, #02, #03, and #05 received antimicrobial therapy for suspected osteomyelitis before AL diagnosis was established. However, apart from case #02, no patient completed a standard course of antibiotic treatment for osteomyelitis, including case #06 who did not receive any antibiotic therapy, and follow-up MRI examinations were not performed. Although musculoskeletal discomfort resolved following the initiation of leukemia-directed therapy and did not recur during follow-up, the potential influence of prior antibiotic exposure and the possibility of concomitant infection remain possible confounders of this study. Therefore, in our cohort, while concurrent osteomyelitis appeared unlikely, it could not be excluded with certainty.

Initial presentation with musculoskeletal pain and osteomyelitis-like symptoms can lead to significant diagnostic and therapeutic delay in children with cancer (33, 34). In our cohort, the maximum delay was 52 days (case #06); however, retrospective studies have reported much longer intervals—in some cases extending up to 1 year (3). Interestingly, according to a large retrospective trial, such delays did not appear to adversely affect patient outcome or overall survival (3). Nonetheless, postponement of appropriate treatment may result in persistent pain and the development of additional hematologic abnormalities, ultimately leading to debilitating symptoms such as fatigue, bleeding diathesis, and recurrent infections, thereby substantially impairing a patient’s clinical condition. The reported cases shared features such as transient clinical response to antibiotic therapy, initially normal hematologic parameters, and multifocal MRI involvement. Although these findings were observed among patients with delayed diagnosis, the study design did not permit any assessment of whether they were associated with or predictive of diagnostic delay.

Several limitations of this study should be acknowledged. Its retrospective observational design and the absence of a comparator group (e.g., patients with confirmed bacterial osteomyelitis, CNO/CRMO, or other inflammatory disorders) prevent the identification of clinical or radiological features that reliably distinguish AL from alternative diagnoses. The findings rather highlight the substantial overlap in clinical presentation and imaging characteristics among these conditions and emphasize the importance of considering leukemia within the differential diagnosis from the earliest stages of evaluation. The study design also does not allow for determination of the true incidence or prevalence of osteomyelitis-like presentations among pediatric acute leukemia (AL) cases, as a systematic analysis of all patients diagnosed with AL during the study period was not performed. As a result, referral bias cannot be excluded. A comprehensive evaluation of all pediatric AL cases, particularly those initially presenting with musculoskeletal symptoms and undergoing MRI prior to the final diagnosis, would be necessary to better define the clinical spectrum and frequency of such presentations.

Moreover, the study cohort was small (*n* = 6) and heterogeneous, including only four cases of BCP-ALL, one AML, and one ALL relapse. The generalizability of the results is therefore limited. Nevertheless, previous case series have similarly presented patients with ALL and AML together (**Table 2**) (10, 13). In fact, despite their different biology, their initial symptoms show a high degree of similarity, and definitive discrimination ultimately relies on immunophenotypic and molecular genetic analysis (2). The inclusion of case #04 may also be considered a source of heterogeneity. However, despite the distinct clinical context of ALL relapse, this patient presented with leading musculoskeletal pain, no hematologic abnormalities, and failure to respond to antibiotics. These features closely mirror those observed in newly diagnosed cases and further illustrate the potential for AL to mimic osteomyelitis, even in the relapse setting.

In addition to the limited number of cases included in our study, the reported MRI findings appear to be largely nonspecific in nature. Therefore, no conclusions can be drawn from this work regarding the predictive value or the diagnostic accuracy of MRI for the early detection of AL, a diagnosis that relies primarily on thorough hematologic evaluation. Nevertheless, our observations suggest that, in some patients with leukemic disease, bone marrow signal abnormalities may precede overt hematologic manifestations. This hypothesis warrants further investigation in larger, controlled studies.

Overall, this study highlights that pediatric AL may initially mimic osteomyelitis clinically, hematologically, and radiologically, thereby possibly contributing to diagnostic delay. Even when osteomyelitis appears the most plausible diagnosis, AL must remain an important differential consideration, particularly in the presence of persistent musculoskeletal pain, negative bacterial tests, inadequate response to antimicrobial therapy, rising LDH or ESR, or newly developing hematologic changes. Finally, although MRI findings may initially appear compatible with osteomyelitis, they should be interpreted with caution given the radiologic convergence between infectious and malignant bone marrow processes. A high level of suspicion for hematological malignancies must be therefore maintained during both treatment and follow-up of such cases.

## Data Availability

The original contributions presented in the study are included in the article/**Supplementary Material**. Further inquiries can be directed to the corresponding author.
