# Supplementary material for: Pediatric acute leukemia mimicking osteomyelitis: clinical and MRI features
Source: Front Oncol. 2026 Jul 14;16:1872357. doi: 10.3389/fonc.2026.1872357 (PMC13407188; doi:10.3389/fonc.2026.1872357)
Supplement: Supplementary file 1 [file Table1.docx]

| Supplementary TABLE S1 Clinical information of patients from Kato et al., Yoshikawa et al., Gomoll et al., and Goto et al. (13, 14, 6, 7) | | | | | | | | | | | | | | |
| --- | --- | --- | --- | --- | --- | --- | --- | --- | --- | --- | --- | --- | --- | --- |
| **Case** | **1 Kato et al.** | **2 Kato et al.** | **3 Kato et al.** | **4 Kato et al.** | **5 Kato et al.** | **6 Kato et al.** | **1 Yoshikawa et al.** | **2 Yoshikawa et al.** | **3 Yoshikawa et al.** | **4 Yoshikawa et al.** | **Gomoll et al.** | **Goto et al.** | **Median** | **Range** |
| **Age (years)** | 13 | 8 | 6 | 5 | 5 | 11 | 3 | 11 | 3 | 2 | 3 | 1 | 5 | (1 - 13) |
| **Gender** | M | M | F | M | F | F | M | M | M | M | M | M | 3 : 1 = M : F | |
| **Chief complaints** | | | | | | | | | | | | | | |
| **Fever** | + | + | + | + | + | + | + | + | + | + | + | + | 100 % + | |
| **Pain** | + | + | + | + | + | + | + | + | + | + | + | + | 100% + | |
| **Laboratory parameters** | | | | | | | | | | | | | | |
| **WBC (/ul)** | 4800 | 10500 | 4900 | 8100 | 6100 | 5700 | 11400 | 51000 | 10400 | 5200 | n.a. | 26900 | 8100 | (4800 - 26900) |
| **Hb (g/dl)** | 13,8 | 13,7 | 13,5 | 11,7 | 10,5 | 13,7 | 12,7 | 11,8 | 13,3 | 11,8 | n.a. | 11,4 | 12,7 | (10,5 - 13,7) |
| **PLT (×103/ul)** | 277 | 305 | 261 | 266 | 238 | 28 | 366 | 423 | 248 | 364 | n.a. | 249 | 266 | (28 - 423) |
| **LDH (IU/l)** | 716 | 1260 | 237 | 2712 | 195 | 195 | 336 | 322 | 613 | 261 | n.a. | 344 | 336 | (195 - 2712) |
| **CRP (mg/dl)** | 2,85 | 9,66 | 2,57 | 7,47 | 4,62 | 0,04 | 1 | 4 | 7,5 | 7,8 | 0.56 | 7,7 | 4,62 | (0,04 - 9,7) |
| **ESR** | n.a. | n.a. | n.a. | n.a. | n.a. | n.a. | n.a. | 130 | n.a. | n.a. | 126 | n.a. | 128 | n.a. |
| **Diagnosis** | BCP-ALL | AML | BCP-ALL | BCP-ALL | BCP-ALL | BCP-ALL | BCP-ALL | BCP-ALL | BCP-ALL | BCP-ALL | BCP-ALL | AML | 6 : 1 = ALL : AML | |
| **Genetics** | 46, XY | 46, XY | 46, XX | 46, XX, gain of 1qb | 46, XY | 46, XX | n.a. | n.a. | n.a. | n.a. | n.a. | 46, XY, t(10;11) (p11.2;q23); FLT3-ID mutated |  | |
| **Outcome** | 1st CR | 2nd CR | 1st CR | 1st CR | 1st Relapse | 1st CR | 1st CR | 1st CR | 1st CR | 1st CR | n.a. | 1st CR |  |  |
| **Follow-up [months]** | 48 | 8 | 55 | 40 | 60 | 5 | 44 | 23 | 104 | 56 |  | 36 | 44 | (5 - 104) |
| **Δt [days]** | n.a. | n.a. | n.a. | n.a. | n.a. | n.a. | 40 | 180 | 27 | 20 | 42 | > 12 | 33,5 | (12 - 180) |
| **MRI Δt [days]** | n.a. | 35 | n.a. | 14 | n.a. | 42 | 28 | 56 | 24 | 14 | > 7 | 12 | 14 | (7 - 56) |
| Abbreviations: BCP-ALL, B-cell precursor acute lymphoblastic leukemia; AML, acute myeloid leukemia; d, day(s); CR, complete remission; CRP, C-reactive protein; ESR, erythrocyte sedimentation rate; F, female; Hb, hemoglobin; LDH, lactate dehydrogenase; M, male; n.a., not available; PLT, Platelet count; WBC, white blood cell count; wk, week(s); Δt, time interval between suspected osteomyelitis (or initial symptoms) and diagnosis of acute leukemia; MRI Δt, time interval between MRI and diagnosis of acute leukemia; +, presence; –, absence. | | | | | | | | | | | | | | |

Supplementary TABLE S2 Laboratory parameters at leukemia diagnosis.

| Patient | # 01 | # 02 | # 03 | # 04 | # 05 | # 06 |
| --- | --- | --- | --- | --- | --- | --- |
| WBC [G/L] | 8.1  (4.5–18.8)^§^ | 2.8 ↓  (4.2–13.3) | 7.1  (4.5–17.0) | 4.4  (3.8–11.9) | 8.7  (4.0 –10.0) | 25.4  (4.8-12.0) |
| ANC [G/L] | 1.77  (>1.5) | 0.59 ↓  (>1.5) | 1.5  (>1.5) | 3.4  (>1.5) | 0.6  (>1.5) | 1.8  (>1.5) |
| Hb [g/dL] | 5.5 ↓  (10–13.7) | 7.9 ↓  (10.9–14.3) | 10.5  (10.3–13.9) | 11.0 ↓  (11.3–14.7) | 8.9 ↓  (12.0–15.0) | 3.6  (11.2-14.6) |
| PLT [G/L] | 151  (159–532) | 247  (177–463) | 282  (165–502) | 219  (178–426) | 258  (150–450) | 10  (180-415) |
| LDH [U/L] | 555  (120–300) | 471  (120–300) | 434  (120–300) | 268  (120–300) | 596  (120–300) | 976  (110-295) |
| Uric acid [mg/dL] | 2.3  (2.4–5.7) | 1.5  (3.4–7) | 1.7  (2.4–5.7) | 4.6  (3.4–7) | 2.1  (2.4–5.7) | 4.9 |
| CRP [mg/L] | 47.6 ↓  (<5.0) | 66.7 ↓  (<5.0) | 13.3  (<5.0) | 57.0  (<5.0) | 76.0  (<5.0) | 17.8  (<5.0) |
| Peripheral blasts [%] | 1 | 15 | 0 | 0 | 2 | 65 |
| ESR was not assessed at leukemia diagnosis.  Abbreviations: ANC, absolute neutrophil count; CRP, C-reactive protein; ESR, erythrocyte sedimentation rate; Hb, Hemoglobin; LDH, lactate dehydrogenase; n.a., not available; PLT, Platelet count; WBC, white blood cell count.  ^§^Age-dependent and gender-dependent normal range is displayed in brackets. | | | | | | |

Supplementary TABLE S3 Level of evidence of references used in Table 2 based on the definitions of the Scottish Intercollegiate Guidelines Network (SIGN) 2017 (34).

| **Level of evidence** | **Definition** | **References** |
| --- | --- | --- |
| **1** | Evidence from meta-analyses, systematic reviews, or RCTs, or large representative population samples with a low to high risk of bias. | 1, 2, 8, 9, 24 |
| **2** | Evidence from high-quality / well-conducted case-control or cohort studies with a very low risk of confounding or bias and a high probability that the relationship is causal. | 3, 4, 22, 23 |
| **3** | Analytic studies without a concurrent comparison group, e.g. before-and-after studies, interrupted time series; nonanalytic studies, e.g. case reports, case series. | 6, 7, 10, 13, 14, 31, 32, 33 |
| **4** | Expert opinion, e.g. editorial commentaries, guidelines without a clear methodology | 5, 18 |
| Abbreviations: RCT, randomized controlled trial; SIGN, Scottish Intercollegiate Guidelines Network. | | |
